# THE GREAT AND THE GRAND: THE ROLES OF GREAT-GRANDPARENTS AND GRANDPARENTS IN MULTIGENERATIONAL FAMILIES

**DOI:** 10.1093/geroni/igz038.1047

**Published:** 2019-11-08

**Authors:** Emily Schuler, Cristina Maria de Souza Brito Dias

**Affiliations:** Universidade Católica de Pernambuco (UNICAP), Recife, Pernambuco, Brazil

## Abstract

The increase of Human Aging has been observed rapidly in the whole world, as it has been in Brazil allowing the experience to live several roles within the family for a longer time. As a consequence, more multigenerational families emerge with a more vertical structure, formed by four or even five generations. While the oldest generation adds another generational role to their life, the one of great-grandparents, the youngest generation is born into an intergenerational network of relationships. There are various questions about the differences in the role of great-grandparents and grandparents, which motivated this present study. Thus, the objective of this study was to understand the roles of great-grandparents and grandparents in the family and their intergenerational repercussions. Four families with for generations, totaling 16 participants. One member of each generation was interviewed, using a specific script, which was afterwards analyzed by the Thematic Content Analysis. The results pointed out that both great-grandparents and grandparents have distinct roles that are constructed around the needs of the family; both figures provide emotional and material support to the family; both roles have transgenerational importance in the transmission of family legacies, which are related to faith, solidarity, education and order. It can also be said that the great-grandparents can be compared to the grandparents of the past, as the grandparents can be assimilated to the parents of older days. It is hoped that this research contributes to the visibility of these two generations and to sensitize professionals about this theme.

